# Silence and Denial in Everyday Life—The Case of Animal Suffering

**DOI:** 10.3390/ani1010186

**Published:** 2011-02-21

**Authors:** Deidre Wicks

**Affiliations:** Department of Sociology and Anthropology, University of Newcastle, NSW 2308, Australia; E-Mail: deidre.wicks@gmail.com

**Keywords:** silence, denial, culture, taboo, passive bystander, emotional management, social censorship, normalization, collective endeavour

## Abstract

**Simple Summary:**

This paper analyses issues implicit in the question: How is it that decent and compassionate people co-exist in silence about widespread animal suffering? The paper explores the complex process of denial which operates at both a personal and societal level to allow people to ‘not see’ and ‘not know’ about the realities of the lives of animals in our world. The paper argues that silence allows animal suffering to exist and flourish at a historically unprecedented level at this time. It goes on to examine the conditions under which silence can be punctured and acknowledgement and action for animals becomes possible.

**Abstract:**

How can we make sense of the fact that we live in a world where good people co-exist in silence about widespread animal suffering. How is it that sites of suffering such as laboratories, factory farms, abattoirs and animal transportation are all around us and yet we ‘do not, in a certain sense, know about them’ [1]. This ‘not knowing’ is one of the most difficult barriers for animal activists who must constantly develop new strategies in an attempt to catch public attention and translate it into action. Recent contributions from the ‘sociology of denial’ have elucidated many of the mechanisms involved in ‘not knowing’ in relation to human atrocities and genocide. In this context, ‘denial’ refers to the maintenance of social worlds in which an undesirable situation is unrecognized, ignored or made to seem normal [2]. These include different types of denial: personal, official and cultural, as well as the process of normalization whereby suffering becomes invisible through routinization, tolerance, accommodation, collusion and cover up. Denial and normalization reflect both personal and collective states where suffering is not acknowledged [3]. In this paper, I will examine insights from the sociology of denial and apply them to human denial and normalization of animal suffering. This will include an examination of denial which is both individual and social and the implications of these insights for theory and practice in the human/animal relationship.

## 1. Introduction

Animal activists and theorists face many problems. Some are theoretical, some purely practical and many are a combination of both. The problem to be addressed in this paper is that we live in a world where good and compassionate people co-exist in silence about widespread animal suffering. How can it be that sites of animal suffering are all around us in factory farms, transportation, rodeos, abattoirs and laboratories and yet we do not, in a certain sense, ‘know about them’ [1]. This state of both ‘knowing’ and at the same time ‘not knowing’ presents particular problems for animal rights/welfare advocates who must strive to speak the unspeakable, show the invisible and illuminate the unknowable to a world which is seemingly determined to behave like the ‘Three Wise Monkeys’ and not see, hear or know the extent of animal suffering in our world. The struggle for intellectuals and activists who act on behalf of animals is to break through the wall of silence and puncture the edifice of denial so that public attention is directed toward suffering and turned into effective action [4]. In what follows, I intend to explore the processes involved in ‘not knowing’ so as to enhance our understanding of the phenomenon and therefore of the capacity for effective analysis and intervention. I will do this by calling on recent developments in the ‘sociology of denial’, which aim to elucidate the mechanisms involved in the mass denial of human to human atrocities and genocide [1,2]. It is my contention that this area of scholarship has much to offer our understanding of the human reluctance to know about and act upon the suffering of animals [5]. Such insights may offer solutions to some key practical and theoretical dilemmas in the field of animal activism.

The extent of animal suffering in the 21st century is not unknown. Through the work of writers like Ruth Harrison [6], Peter Singer [7,8,9], Christine Townend [10] and the many writers who have followed, a picture of widespread, sustained and endemic animal suffering exists, much of it deliberately inflicted by our hands. The sites of suffering are endless but tend to be focused on laboratories, farms (especially factory-farms), long-haul transport, abattoirs, hunting and habitat destruction for commercial use. In purely numerical terms, perhaps the greatest suffering is inflicted on animals through the transformation involved in turning their bodies into food. This is now happening on a massive industrial scale. It is illustrated in the fact that, in 2003, the United States became the first nation to raise more than ten billion farmed animals in one year [11]. That such increases in production have come at a terrible cost to animals has now been extensively documented and is not the immediate subject of this paper. It is, however, important to establish that these facts are not a secret. The information is readily available to those who make the slightest effort to find out. Occasionally, the information is even presented uninvited, as when reports appear in the mass media regarding animal escapes from the slaughterhouse, numbers of animals killed in laboratory research or protests against raising hens in battery cages. When these reports do appear, there is often very little follow up. The report appears in isolation, at most might have a follow-up letter to the editor the next day, and then sinks like a stone. There is a brief moment of ‘knowing’ about some aspect of animal suffering which is then replaced by, or co-exists with, forgetting and ‘not knowing’. This process is referred to as denial. This concept helps us understand how we can be aware and unaware of something at the same time [2]. A fuller understanding of the process of denial is clearly crucial for any person or group seeking positive change in this area.

Denial can be defined as: ‘the maintenance of social worlds in which an undesirable situation (event, condition, phenomenon) is unrecognized, ignored or made to seem normal’ [3]. Denial is necessary only when knowledge which is confronting and liable to cause cognitive and emotional distress becomes widely and irreversibly disseminated, in other words, when the secret gets out. It has most commonly been studied from the psychological perspective of the individual, yet sociologists point out that what individuals choose to ignore or to notice must be understood in the context of social norms as well as the wider political and economic context [12]. In the case of animal suffering, denial works hand in hand with another powerful phenomenon—silence. Indeed, the groundbreaking writer in this field has argued that silence is the most public form of denial [2]. He has argued further that a sociological approach allows us to see denial as a social process which is a collective endeavour and which involves a collaborative effort [2]. This provides us with an entry point for understanding the silence that surrounds the reality of animal suffering. For if silence and denial is social and collaborative, it must contain discernible patterns and dynamics. To understand this is to become aware of strengths, weaknesses and points of possibility for breaking silence and widening public discourse.

## 2. Cultural Denial, Cultural Censorship and Emotional Management

Stanley Cohen has argued that denial can be personal, official and cultural [3]. Cultural denial, which is of most relevance for this discussion, is neither wholly private nor officially organized by the state. Cohen argues that whole societies may slip into collective denial without either public sanctions or overt methods of control. Without being told what to think, societies can arrive at unwritten agreements about what can be known and acknowledged. In order for this to be feasible, there must be a shared cultural vocabulary on which to draw. This shared vocabulary represents a commitment between people (couples, families, entire populations) to support and collude in each others' denials [3]. Without conscious negotiation, people know which facts are better not noticed and which trouble spots to avoid. For instance, people do not consciously repress mention of slaughterhouses when they are guests at a BBQ or dinner party where meat is being served. At the same time, they call on a common vocabulary to discuss the ‘tenderness’ of the meat (not how young the animal was) and the ‘juiciness’ of the steak (not how much blood and lymph fluid it contains). There is an unspoken, indeed unconscious agreement that such references would be bad manners or bad taste. This is why the mere presence of a vegetarian at a dinner table can make people uncomfortable. Their presence raises into consciousness all those ideas and images so carefully ‘not known’ and ‘not seen’. The discomfort felt by others at the table can lead to either aggression or self-justification toward the vegetarian. This, however, is not seen as a breach of good manners because it is the vegetarian who is the outsider, the threat to social cohesion. The presence of a self-declared vegetarian at the table punctures the carefully constructed edifice of personal and cultural denial concerning the suffering of the animal that was turned into meat for the meal.

Robin Sheriff goes further in her analysis when she describes silence as social censorship. She is concerned with a particular kind of silence through which ‘various forms of power may be partly, although often incompletely concealed, denied or naturalized’ [13]. She states further that various sorts of patterned silences play roles in constructing the shape of social and political life. We can see both these processes operating in the way that the silences surrounding animal's place in the moral universe allow the ongoing and lucrative mistreatment of animals to continue at the same time that the shape of social and political life is reinforced by the very forms of exploitation it denies. Our society and polity, for instance, is shaped by meat eating in terms of health issues, greenhouse gas emissions, land use patterns, and the power of large, often multi-national, agri-business corporations to influence the policies of governments (such as live-export of cattle and land clearing). At the same time, both private and public life is shaped by the collective denial entailed in the living reality of breeding, housing, transporting and finally killing huge numbers of animals, often in appalling conditions on a daily basis.

In this situation, denial operates to protect people from unpleasant feelings, described by Kari Norgaard (in relation to her study on climate change) as feelings of helplessness and guilt as well as the emotion of fear of ‘being a bad person’ [12]. It is silence that allows denial to work in a way which is functional for the satisfaction of personal desires without the need of having to experience unpleasant emotions. Denial then becomes a way to hold unpleasant information at a distance and so acts as a form of emotional management. Clearly, such denial of one's feelings is psychologically exhausting. Zerubavel also points out that conspiracies of silence may trigger feelings of loneliness among those who refuse to partake in the conspiracy [2]. The discrepancy between what an individual notices compared with what one's peers choose to ignore can lead to feelings of deep isolation. Where open communication brings us closer, silence brings distance between us. When this distancing from unpleasant information is a collective enterprise, it can be seen as the social organization of denial [2]. The costs then also become social. Ignoring ‘the elephant in the room’ requires a serious collaborative effort on everyone's part and may become socially exhausting. It is also likely to generate varying amounts of tension. The deeper the silence, the thicker the tension that builds around it is likely to be. This tension builds between co-conspirators as well as between conspirators and those who actively notice and acknowledge. In addition to social tension, silence is also morally corrosive, as it inevitably opens up possibilities for abuse. It is well known that silence and secrecy is the perpetrators' main weapon of advantage. This, however, is a weapon which is not wielded by the perpetrator alone. We all know the saying ‘silence is consent’. By remaining silent about cruelty or suffering we help to normalize it by implicitly encouraging potential offenders to regard it as morally acceptable. Indeed, breaking the silence is considered by many to be a ‘moral act par excellence’ [2]. More often than not, individuals do not break the silence and therefore become bystanders. This concept is worthy of closer examination.

## 3. The Passive Bystander

After World War II, many scholars turned their attention to the vexed question of how the good and highly cultured people of Germany could have allowed such terrible acts of genocide to be perpetrated in their midst. The sociologist Everett C. Hughes posed the key question in a seminal publication in 1962 when he asked:

How could these millions of ordinary people live in the midst of such cruelty and murder without a general uprising against it and against the people who did it? … How and where could there be found in a modern civilized country the several hundred thousand men and women capable of such work? How were these people so far released from the inhibitions of civilized life as to be able to imagine let alone perform, the ferocious, obscene, and perverse actions which they did imagine and perform [14]?

Everett points out that there are two orders of question here. The first concerns those who did the work, while the second concerns those who allowed it to happen. In an important sense, though, they are both connected in that the silence of one group enabled the actions of the others. This leads Hughes to raise the crucial question: under what circumstances will good people let others get away with such actions? A related question is raised later when he states: ‘This must make us ask under what conditions the will to know and to discuss is strong, determined and effective’ [14]. These remain important questions for those of us today who work in fields where the bystanders to suffering are as crucial as the perpetrators. This necessarily includes bystanders to the suffering of animals.

Writing many decades later on the same subject, Zerubavel makes the point that silent bystanders act as enablers because watching others ignore something encourages others to deny its presence. It is much more difficult to trust one's own perception when no one else around appears to notice what you do: ‘The discrepancy between others' apparent inability to notice it and one's own sensory experience creates a sense of ambiguity that further increases the likelihood that one would ultimately succumb to the social pressure and opt for denial’ [2]. This pressure is further compounded when the number of bystanders is large. If you are in a minority of one or two, it is even more difficult to maintain confidence in one's own perception and knowledge of the truth. The other factor which will affect the resilience of the silence is the length of time it is maintained. Rather than the likelihood that a silence would be interrupted the longer that it lasts, it instead becomes stronger as time goes on. Like any other form of denial, silence is self-reinforcing. This made it so much harder for the ‘second wave’ pioneers of animal rights to speak out against the animal cruelty involved in factory farming, which was the ‘elephant in the room’ of the post second world-war economic and industrial boom which nobody wanted to notice. If silence is self-reinforcing, what actually can be done to bring conspiracies of silence to an end? This is a question to which I will return in the last section of the paper, but first it is necessary to deepen our understanding of the social structure of denial by explicating its rules.

## 4. The Rules of Denial

In his systematic attempt to analyze the social nature of silence, Zerubavel explores the cognitive and behavioural skills that enable us to participate in conspiracies of silence. He calls these skills the rules of denial—they make denial possible. I have adapted his schema and applied it to the conspiracies of silence around animal suffering. Zerubavel argues that conspiracies of silence begin with noticing and not noticing.

### 4.1. Attention and Culture

While we all have specific physiological limitations on what we can see and hear and smell, what we notice from the surrounding background and what we ignore is only partly explained by nature. It is possible, for instance, for two people to be present at an event and report very different versions of the same thing. The ‘non natural’, clearly social foundations of the way we pay attention to things are evident from the way attention habits differ between social groups. Many Chinese, for instance, will find the presence of caged wild animals in market places unremarkable, whereas a Western tourist happening on such a scene is likely to notice it and find it very remarkable and/or distressing. The social underpinnings of what we notice and what we ignore are also evident from the fact that these things shift historically. 80 years ago people in Sydney would have been unlikely to notice a hungry dog in the street (unless it was a threat). These days such a dog would be noticed, reported, and impounded immediately. This theme is reminiscent of some of the work undertaken by the sociologist Norbert Elias [15]. Elias undertook a theoretical exposition of historical change which is based on the link between long-term structural development of societies and changes in people's behaviour concerning such ‘natural’ functions as preparing and eating food, washing, spitting, and defecating. While these may appear to be trivial behaviours on which to focus, it is precisely the unavoidable necessity of the tasks that makes any changes in the way they are performed visible as social changes. Through the lens provided by Elias, it is possible to ‘see’ great changes in the way food is prepared, presented and, by implication, noticed. It has, for instance, entailed the removal of the obvious signs of the living and dead animal from public view. Whereas in the 1950s people did not notice whole carcasses hanging in butcher's shops, or the calves' and pigs' heads in the window, now it would be noticed and probably considered ‘tasteless’.

As mores of noticing and attention change historically, so moral horizons also shift. Where once people of different colour, gender, and sexual orientation were considered to have a different moral weight, it would now be inconceivable to think and talk about people in this way. The last thirty years have also seen attention and moral concern shift toward other species in a way that did not seem possible decades ago. While it is still considered a ‘minority concern’, the animal rights movement has grown beyond expectations [9].

### 4.2. Learning to Ignore

In the same way that attention and noticing are social acts that are learnt in specific places, communities and times, so too is the act of not noticing or ignoring. Zerubavel makes the point that ignoring something is more than failing to notice it. It is often the result of some pressure to disregard it. In fact we normally internalize traditions of paying attention as part of our socialization [2].

By watching others (parents, teachers, peers) ignoring certain things, we learn to ignore them as well. An obvious instance of this is the way that children are trained to ignore the reality of meat. They are taught to call things by certain names like ‘chops’, ‘bacon’, ‘roast’, and ‘cutlet’. When the fateful day arrives when the child finally asks ‘What is a chop made out of?’ they are inevitably told a version of the truth which they understand to mean, ‘Do not ask about this’. Do not notice that this is anything but a ‘chop’. And so the training in denial begins.

This is particularly evident in the tacit social rules that determine what we consider irrelevant in both personal and professional life. It is important to note that the act of separating the ‘relevant’ from the ‘irrelevant’ is an act performed by members of social communities who have been socialized to focus on certain parts or aspects of situations while systematically ignoring others [2]. In this way, it is also implicitly a political act. This is clear when we either notice or do not notice poverty, homelessness and inequality, but it also applies in relation to what we notice and ignore concerning animals. How easy is it not to notice the ducks which hang in the windows of Chinese BBQ shops and restaurants? How easy not to see that their heads are completely intact? How easy not to ask the question: ‘How did they die’? People are not queued up outside staring, shaking their heads and wondering out loud how they were killed. No, they see the dead, cooked bodies as potential food, the manner of their death as irrelevant. In the same way, it is expected that when a whole fish is presented on a plate that we will ignore its face and clouded eyes. Any comments or jokes about its face are recognized as being on the borderline of good taste. This leads us to the next rule of denial—that of tact and taboo.

### 4.3. Tact and Taboo

Tact and taboo are integrated social processes which create conditions that are conducive to the functioning of denial around animal suffering. Characterized by a strong emphasis on avoidance, taboos frequently manifest themselves in the form of strict prohibitions against looking, listening or saying. Those who defy or ignore these prohibitions are universally regarded as social deviants. This has been the case for animal rights activists who are still seen to be on the borderline of mainstream society because they speak what should be neither said nor heard. Their message is potentially offensive at every level from the personal (how we as individuals treat animals) to the challenge offered to large corporations (what they do to animals in order to make money out of them). The message of animal rights and animal welfare has also been seen as taboo because in the past it was seen to run counter to Enlightenment discourses of rationality. Any discussion on animal suffering was seen to be based on ‘emotion’ (which was necessarily bad) and that most powerful epithet ‘anthropomorphism’. In recent decades, researchers have worked to provide scientific evidence for claims concerning the cognitive and emotional capacities of animals, including their capacity for suffering [16,17,18]. Discussions, writings, debates, and conferences on animal issues can now be seen to be crossing a barrier (in Western developed countries and in some developing countries) from irrelevant and illegitimate to relevant and legitimate in both popular and academic circles.

A form of verbal avoidance that contains elements of both taboo and tact is the use of euphemisms. The use of such words is rife in the intersection of the human treatment of animals and is essential for the maintenance of denial of the reality of animal suffering. Euphemisms take the sting out of truth and aid and abet the continuation of denial where complete silence is not possible. Indeed, euphemisms may help uncover conspiracies of silence by highlighting what it is that is considered unmentionable. A bizarre example of this occurs in country towns in the West of Ireland (and perhaps elsewhere) where the abattoir or slaughterhouse is referred to as ‘the factory’ [19]. On reflection, it can be seen that the word emphasizes the ‘making’ of something rather than the annihilation of living creatures. A farmer might say: ‘If that cow doesn't produce a calf this year she will have to go to the factory’. There presumably she will get a job making meat. Interestingly, the only public reference to the actual killing of animals is the derogatory name given by locals to members of the travelling community who were known as ‘knackers’ (horse killers). In Australia, we have endless euphemisms for the cruel practices we inflict on animals. Terms such as mulesing, crutching, beak trimming along with ‘porcine stress syndrome’ and ‘caged layer fatigue’ are designed to hide rather than reveal the reality of causing pain and death to animals, such as the cutting and slicing of living flesh that takes place without anesthetic. In another way, even neutral sounding words like ‘livestock’ and ‘cattle’ objectify animals by presenting them as a plural entity at the same time as denoting them as property. Words, then, are important in either maintaining or revealing conspiracies of silence. The challenge for those of us inevitably living within conspiracies of silence is to recognize the euphemisms when we come across them. Whether or not we decide to expose them will depend on a great many things, including the social sensitivity known as ‘tact’.

Tact can be seen as a milder form of taboo. It falls in the category of ‘it might be considered rude’ rather than ‘it is strictly forbidden’. While it is subtle, it is also powerful. It operates through the sanction of embarrassment rather than through fear. While it may be seen as being weaker than taboo, it is also more insidious in that it does not have the cachet of opposition as resistance but rather as ignorance or social ineptitude. Being accused of being ‘tactless’ is both personally hurtful and can also call up images of social ineptitude. Raising issues of animal suffering in most social situations can be viewed as ‘tactless’ because many such situations involve some form of animal exploitation. If the issue is raised when someone is wearing fur or leather, that is considered ‘tactless’. If it is raised in a restaurant, hospital, zoo or circus, that is also ‘tactless’. In fact, tact can be seen as a highly effective sanction for silencing comments and discussion around animal suffering.

## 5. The Politics of Denial

Having dealt with some of the sociological dimensions of denial, this section examines denial in the wider political and economic context. It is clear that in order to discuss the politics of denial, it is necessary to move beyond the behaviour of individuals and groups to look at the institutional processes and structures which permit and promote the entry of some issues on the political agenda while inhibiting or preventing others. In the pluralist view of power, it is posited that decisions are made on the basis of actual, observable conflict. This includes decisions concerning what gets attention, what gets discussed (privately and in the media) and what gets ignored. This view has been criticized by the sociologist Steven Lukes who put forward a ‘three dimensional’ view of power which allows for the possibility that power may be operating where there is no discernible or overt conflict but a *latent* conflict of interest. He posed the question: ‘Indeed, is it not the supreme exercise of power to get another or others to have the desires you want them to have—that is, to secure their compliance by controlling their thoughts and desires?’ [20]. Crucially, Lukes went on to point out that this kind of control can take relatively mundane forms such as the control of information, the mass media and through the process of socialization. Zerubavel picks up from this point when he states that power involves the ability to control the scope of others' attention [2]. Apart from early socialization by parents, one of the earliest examples of this control is the scope of the school syllabus which is taught by teachers. Knowledge is parceled up into subjects, with animals relegated to sample and experiment status in biology. Nowhere, for instance, is the rich history of animal welfare taught in schools. This situation continues into higher education where the subject of animals has only recently been admitted into some schools of philosophy and law. Animals are most commonly found in universities as objects of experimentation. When anthropologist Barbara Noske was preparing to undertake her Ph.D., she was advised not to study the human/animal relationship as it did not ‘fit’ the discipline. In fact, it did not ‘fit’ anywhere. It took great determination to produce the thesis which eventually became the groundbreaking book *Beyond Boundaries* [21,22].

### 5.1. Setting the Agenda

The other obvious way that power operates to silence certain issues is by controlling the agenda. This happens within organizations where certain items can be excluded from ever emerging for discussion and debate. Such was the case with the main animal welfare body, the RSPCA, where for years (now changed), farming practices were outside the organization's remit and therefore outside any legislative framework. Until recently, this allowed factory farming practices to remain immune from legislative sanction and indeed from public scrutiny until other organizations such as Animal Liberation emerged to throw light on this hidden area of animal suffering. Agenda-setting also takes place at the national level. When successive governments place the live export of sheep and cattle high on our list of trade priorities, they are essentially denying that the transportation of animals by ship to the Middle East is inherently cruel and wasteful. They do not talk about the fact that in 2008 alone, 40,241 sheep died on boats on their way to the Middle East [23]. When secret reports and/or film footage reveal appalling cruelty in Middle Eastern slaughterhouses, government bodies respond with what Cohen has called ‘interpretive’ denial: ‘It was not meant to be like this, this is an exception’ [3]. In this way, the overall structure of denial remains intact.

### 5.2. The Mass Media

The ultimate attention-grabbing power resides in the mass media which determines what is eventually displayed on our collective radar screen. Zerubavel points out that the media may not be so successful in telling us what to think, but they are stunningly successful in telling us what to think about [2]. In addition, by deciding which issues and events make headlines and become lead stories, they also determine their public relevance. The media also keep certain things out of our awareness by simply not covering them. Cohen draws attention to the research which shows that, all things being equal, the most important determinant of selection is whether the story is already a story [3]. Understanding this, PETA have been very successful with their strategy of staging an outrageous stunt that becomes news so that they can then follow up with letters and interviews to defend their campaign and outline the particular instance of animal suffering that they wish to highlight [24]. As well as determining which stories will be covered, the media also determine how long an item will remain ‘news’. In 1999, the Newcastle branch of Animal Liberation (Australia) combined with the local branch of the Maritime Union of Australia (MUA) to campaign successfully against a proposal to export live cattle from the Newcastle Port. At the time, both the local newspaper and television station ran footage from secret filming which had taken place in the Middle East by some Irish members from Compassion in World Farming. The footage showed brutality and cruelty to cows during transportation and slaughter. For a time, it became ‘news’ with the result that the proposal was dropped. But the ‘news’ did not extend to the issue of live export in general and after a short period of time the issue disappeared from view, despite numerous reports since by Animals Australia, the general silence over this practice continues [25].

The media is not just successful in guiding what we think about, but also *the way* we think about it. The presentation of news items is marked by the lack of connections made between items. For instance, there is occasionally a news item regarding the escape of animals from a holding pen on the way to slaughter [26]. Media and popular support invariably fall behind the animals who briefly become heroes and often end up in an animal sanctuary to live out their lives. There is, however, no follow-up on the companions of the escapees or any description of their fate. This allows the general population a moment of ‘feeling good’ about the escapees while remaining in denial about the fate of the vast majority of animals who do not escape. When animal cruelty is presented in the media, it usually falls into three main categories. First, as an anomaly, that is, as an act by a disturbed or mad individual. Second, if the cruelty is more widespread, it will focus on the fact that it happened in an atypical ‘bad’ farm or ship where regulations have been flouted. Third, it will be concerned with something that happened in a ‘foreign’ place such as Moon Bears in China or Rhinos in Kenya. Animal cruelty and suffering is rarely (if ever) presented as part of our ‘normal’ daily life. There have been, for instance, no exposes by the mainstream media on the reality for dairy cows behind milk production or the reality behind the increased production of leather sofas and car seat covers. This leads to the final area to be examined in relation to denial and animal suffering: the economics of denial.

### 5.3. Economics of Denial

The key point and the obvious one to be made about the economics of denial is that the most endemic and numerically significant forms of animal suffering are also those which are supported by large and powerful economic interests. These interests have every reason to co-operate in the maintenance of a public denial concerning the reality of factory farming. Enormous fortunes are made in animal agriculture and the top companies regularly deliver huge returns to their investors. In Australia, for instance, 80 per cent of meat chickens are supplied by three companies. The largest, Inghams Enterprises Pty Ltd has over 8,000 employees and operates in all Australian states. In 2007 its group turnover exceeded AUD$1.6 billion [27]. Another example from the US, Tyson Foods, will further illustrate the point. An AUD$10,000 purchase of Tyson stock in 1975 would have grown to an AUD$6.96 million holding in the company by 2005 [11]. And yet, as profits have grown so enormously, the share to the farmer has dropped. In Britain, for instance, for every pound spent in the shop now on food, just nine pence goes back to the farmer and rural communities, whereas 50 years ago that figure was 50 to 60 pence [28]. One of the reasons for this is the low prices paid for agricultural products. While the cost of housing and cars has risen enormously over the past fifty years, the prices paid for animal products have lagged far behind the overall inflation rate. In fact, when inflation is taken in to account, animal products cost less today than they ever have before [11]. The main reason for this is the efficiencies that have been introduced into animal agriculture. Fewer workers tend vastly more animals and the animals themselves are far more productive due to selective breeding and the widespread use of antibiotics and other pharmaceuticals [29]. As we now know (and try not to know), these efficiencies have come at a terrible cost to both humans (through anti-biotic resistance) and farm animals who now lead lives of abject misery.

## 6. Breaking the Silence and Puncturing Denial

Given the embeddedness of the denial of both the social and personal and the pervasiveness of the silence around issues of animal cruelty, we need to ask: What are the conditions under which information is acknowledged and acted upon? Cohen puts the dilemma succinctly when he expresses the task as ‘how to transform ignorance into information, information into knowledge, knowledge into acknowledgement and finally, acknowledgement into action’ [3]. Cohen works from the premise that denial, in the sense of shutting out awareness of others' suffering, is the normal state of affairs. Far from having to be pushed into accepting reality, people have to be dragged out of their (comfortable) reality [3]. By taking the state of denial as normal rather than aberrant, it is possible to see acknowledgement as the active and uncommon opposite of denial which has to be worked for in several domains. Following Cohen, these domains are: the cognitive (knowing what is happening and retaining the information in a zone of awareness); the emotional (experiencing feelings of empathy, outrage, shame, compassion); the moral (believing this is wrong, this must be changed). To be effective, appeals must reach the threshold of: ‘I cannot keep silent about this any longer; I have to do something’. Finally, for silence and denial to be countered on a mass basis, it must be countered in the domain of culture.

Breaking a conspiracy of silence involves making the open secret (the ‘elephant in the room’) part of the public discourse. This must include public challenges to the more meretricious vocabularies of denial—the euphemisms which hide animal suffering. Like silence itself, breaking it is a collaborative effort that involves an entire social system. In order to counteract the group pressure to keep the silence, it is essential to build the weight of numbers. As more people join the silence breaker, the dynamics of the situation shift until it reaches a ‘tipping point’ where ‘the increasing social pressure on the remaining conspirators to also acknowledge the elephant's presence eventually overrides the social pressure to keep denying it’ [2]. Until this stage is reached, however, the situation for silence breakers is not easy. There is widespread agreement in the literature that it is common for silence breakers to be ridiculed, vilified and often ostracized [2,3,29]. Of course, it helps if the silence breaker is well known or is in a powerful position.

In addition to speaking out and hearing the message, the cultural channels should visibly be in place: to validate the sense that something *can* be done, inform you *what* this something is and enable you to do it. There must be easily recognized paths between general support and good intentions and a starting point for what the professionals call ‘consequential social action’ [3]. For instance, a campaign to expose and create opposition to the factory farming of chickens or pigs must include clear channels for action which cover the spectrum from making a donation, to eating less meat, to writing a letter, or joining an organization and becoming part of a social movement.

Finally, studies of those people who punctured denial and became ‘rescuers’ in times of great danger have revealed some generalized principles that are of relevance here. A recent, (retrospective) study of people who were willing to risk their lives by rescuing Jews in Occupied Europe has confounded rational choice theorists when it found no evidence that rescuers were motivated by any conscious calculation of costs and benefits. Rather, their altruism resulted from a particular cognitive outlook—a sense of self as part of a common humanity rather than being tied to specific interests of family, community or country [3]. Can this form of ‘inclusivity’ be enhanced through education and legislation? These are issues that are germane to animal rights activists and deserve further research.

## 7. Conclusions

This paper has analyzed issues implicit in the question: How is it that otherwise good and compassionate people co-exist in silence about widespread, animal suffering? How can it be that the sites of suffering are present in our midst in the form of factory farms, animal transportation, rodeos, abattoirs, and research laboratories, and yet the majority of people live in a way that allows them to ‘not know’ about the suffering that happens every hour of every day in these places? In exploring this question, I have examined the mechanisms involved in the maintenance of silence and denial concerning animal suffering at both an individual and societal level. This analysis has been made possible by applying insights from the ‘sociology of denial’ which is concerned solely with human atrocities and suffering. This tradition, while developed for understanding and countering human to human atrocities, has an important contribution to make for enhancing our understanding of the silence surrounding animal suffering. It is clear from the exposition presented in this paper that the move from denial to acknowledgement is complex and not easily achieved. It certainly requires more than simply providing information regarding suffering. Indeed, Cohen [3] argues that acknowledgement must be achieved in all the domains involved in denial. These are: the cognitive, the emotional, the moral, and the cultural. Taking insights also from the ground-breaking work on silence by Zerubavel [2], I have focused on the centrality of the domain of culture for countering the denial of animal suffering. This is productive because both silence and denial are collective, social phenomena which are reinforced through the operation of power and which themselves shape social and political life.

While the focus in this paper has been an explication of the mechanisms and processes of denial, I have also examined the conditions under which acknowledgement of suffering and ‘consequential social action’ becomes possible. Key to this is the refusal to participate in the euphemisms which hide animal suffering and the necessity to join the weight of numbers of the silence breakers. In addition, this paper has explored two other factors that further develop our understanding. The first concerns the importance of having clear paths that link acknowledgement of suffering around a particular form of animal cruelty (e.g., battery egg production) with the necessary tools for opposition. This is essential so that good intentions can be translated into effective public, political and economic action. In addition, the paper has looked at the issue of altruism and the fact that ‘rescuers’ commonly have a sense of self as part of common humanity rather than being tied to the specific interests of family, community, or country. Evidence and understanding of the existence of altruism will be crucial for work aimed at the alleviation of animal suffering. Just as important will be our ability to enhance ‘inclusivity’ to the point where other animals, besides our species, are automatically included. For this task, the tradition of the sociology of denial has a special role.

## References

[1] Coetzee J.M. (1999). The Lives of Animals.

[2] Zerubavel E. (2006). The Elephant in the Room: Silence and Denial in Everyday Life.

[3] Cohen S. (2001). States of Denial Knowing about Atrocities and Suffering.

[4] Patterson C. (2002). Eternal Treblinka: Our Treatment of Animals and the Holocaust.

[5] Wicks D., Germov J., Williams L. (2008). Humans, Food and Other Animals: The Vegetarian Option. A Sociology of Food and Nutrition The Social Appetite.

[6] Harrison R. (1964). Animal Machines.

[7] Singer P. (1975). Animal Liberation: A New Ethics for Our Treatment of Animals.

[8] Singer P., Mason J. (2006). The Ethics of What We Eat.

[9] Singer P. (2008). Defence of Animals: The Second Wave.

[10] Townend C. (1980). Defence of Living Things.

[11] Marcus E. (2005). Meat Market.

[12] Norgaard K.M. (2006). People Want to Protect Themselves a Little Bit: Emotions, Denial, and Social Movement Nonparticipation. Sociol. Inquiry.

[13] Sheriff R.E. (2000). Exposing Silence as Cultural Censorship: A Brazilian Case. Am. Anthropol..

[14] Hughes E.C. (1962). Good People and Dirty Work. Soc. Probs..

[15] Elias N. (2000). The Civilizing Process.

[16] Dawkins M.S., Singer P. (2008). The Scientific Basis for Assessing Suffering in Animals. Defence of Animals: The Second Wave.

[17] Bekoff M. (2007). The Emotional Lives of Animals.

[18] Pepperberg I.M. (2000). The Alex Studies.

[19] Wicks D. (2005). Private communication.

[20] Lukes S. (1974). Power. A Radical View.

[21] Noske B. (1989). Humans and Other Animals.

[22] Noske B. (1997). Beyond Boundaries: Humans and Animals.

[23] RSPCA (2010). Live Exports. http://www.rspca.org.au/how-you-can-help/campaigns/liveexports/history-of-disasters-at-sea.html/.

[24] Dawn K., Singer P. (2008). Moving the Media: From Foes, or Indifferent Strangers, to Friends. Defence of Animals: The Second Wave.

[25] Wicks D. (1999). Private communication.

[26] Webster S. (2009). Cow Uses Gorilla Tactics. The Sun-Herald.

[27] Voiceless (2008). From Nest to Nugget—An Expose of Australia's Chicken Factories.

[28] Stevenson P. (2002). The Economics of Factory Farming.

[29] Weber K. (2009). Food Safety Consequences of Factory Farms.

[30] Morrison E.W., Milliken F.J. (2000). Organizational Silence: A Barrier to Change and Development in a Pluralistic World. Acad. Manag. Rev..

